# IL-6 is involved in thoracic ossification of the ligamentum flavum

**DOI:** 10.1371/journal.pone.0272357

**Published:** 2022-07-29

**Authors:** Ann Yehong Huang, Li Shu, Zhongqiang Chen, Chi Zhang

**Affiliations:** 1 Department of Biochemistry, University of Texas Southwestern Medical Center, Dallas, Texas, United States of America; 2 Department of Orthopedics, Peking University International Hospital, Beijing, China; 3 Department of Orthopedics, Peking University Third Hospital, Haidian District, Beijing, China; 4 Biomedical Engineering Department, Peking University, Beijing, China; Augusta University, UNITED STATES

## Abstract

Thoracic ossification of the ligamentum flavum (TOLF) is a heterotopic ossification of spinal ligaments. TOLF is the major cause of thoracic spinal canal stenosis and myelopathy, and its underlying mechanisms are not clear. Bone formation is a complex developmental process involving the differentiation of mesenchymal stem cells to osteoblasts, and regulated by BMP2, RUNX2, Osterix (OSX), etc. In this study, we continue to further characterize properties of TOLF. Our immunohistochemistry experiments showed that expressions of osteoblastic factors such as BMP2 and RUNX2 increased in TOLF. According to flow cytometry analysis the proportion of S phase of cell cycle in primary TOLF cells was 9% higher than the control. Alizarin red staining and ALP staining observations were consistent with immunohistochemistry results. It was also observed that inflammatory cytokine IL-6 level dramatically increased in the culture supernatant of primary TOLF cells. We propose the hypothesis that IL-6 is involved in TOLF. To testify the hypothesis, we examined the effect of IL-6. Our results showed that IL-6 was able to activate expressions of osteoblastic factors such as BMP2, RUNX2, OSX, OCN and ALP, and that expressions of cell proliferation factors cyclin D1 and cyclin C increased in the presence of IL-6. Moreover, IL-6-induced BMP2 expression was inhibited by p38 inhibitor SB203580, indicating that IL-6 regulated the osteogenic BMP2 activation through p38 MAPK pathway. These data suggest that IL-6 is involved in TOLF.

## Introduction

Thoracic ossification of the ligamentum flavum (TOLF) is a heterotopic ossification of spinal ligaments, and TOLF affects people of the East Asian mostly [1,2]. The lower thoracic spine is more likely to be affected in TOLF. Thoracic spinal canal stenosis and myelopathy have been indicated to be caused by TOLF [3–6]. The etiology of TOLF has been explored by different research groups, and their findings demonstrated that some factors may be associated with TOLF, including inflammatory factors [2,7], mechanical effects [8,9], and genetic factors [10,11]. However, the mechanism of TOLF progression is not clear yet.

Bone formation is a complex developmental process involving the differentiation of mesenchymal stem cells to osteoblasts [12]. Osteoblast differentiation is mediated by some important transcription factors, including Indian hedgehog, RUNX2 and Osterix (OSX) [12]. OSX as an osteoblast-specific transcription factor is required for bone formation and osteoblast differentiation [13,14]. OSX was originally discovered as a bone morphogenetic protein-2 (BMP2) inducible gene in mesenchymal stem cells, and OSX knock-out mice lack bone formation [13]. Based on some clinical studies and animal model research, there has been an association between the inflammation and new bone formation. For example, Ankylosing spondylitis as a chronic inflammatory autoimmune disease tends to affect the axial joints, and is characterized by pathological new bone formation [15,16]. Heterotopic ossifications and some diseases with extra new bone formation happen to be preceded by an inflammatory process [15,16]. More and more attention has been paid to the possible role of inflammatory factors on new bone formation. It was demonstrated that a herniated intervertebral discs (IVD) released inflammatory cytokines, such as interleukin-6 (IL-6), nitric oxide, and prostaglandin E2, while interleukin-1a (IL-1a) and tumor necrosis factor-a (TNF-a) were not detected in the culture media of either the herniated or control discs [17]. It implied that inflammatory cytokine IL-6 may be involved in disc degeneration and radiculopathy. In continued studies by the same research group, ligamentum flavum and herniated lumbar IVD tissues were obtained, and ligamentum flavum cell cultures were treated with disk supernatant from herniated IVDs. There was an increase in mRNA expression of osteoblastic biomarker gene osteocalcin (OCN), and positive stain was observed for another osteoblastic biomarker alkaline phosphatase (ALP) in ligamentum flavum cultures with disk supernatant. It suggested that cytokine and related factor secretions from herniated IVDs might affect the ligamentum flavum over time in terms of cellular proliferation, matrix synthesis as well as osteogenesis, eventually leading to possible ligamentum flavum hypertrophy and ossification [17,18].

It has been revealed that IL-6 acts indirectly on osteoclastogenesis by stimulating the release of RANKL by cells within bone tissues such as osteoblasts [19]. IL-6 may participate in the pathogenesis of rheumatoid arthritis (RA), including osteoporosis, not only in the inflamed joints but also in the whole body [20–23]. In some studies, the effects of IL-6 on JAK/STAT3 and SHP2/ERK signal transduction pathways have been explored in osteoblasts, though it is still controversial whether the differentiation is enhanced by IL-6 [20,24]. Some researchers have observed that the PI3K/Akt pathway triggered by IL-6 plays important roles in various different cells [25–29].

Our recent study has showed that the inflammation factor TNF-α is involved in ossified ligamentum flavum in TOLF [2]. In this study, we continue to further characterize properties of TOLF. We examined the effect of IL-6 on the ligamentum ossification process. Our observations suggest that IL-6 is involved in TOLF.

## Materials and methods

### Patient samples

Patients were recruited from Orthopedics Department in Peking University Third Hospital between August 2015 and May 2017. This work was approved by the Ethics Committee of Peking University Third Hospital, and complied with the Declaration of Helsinki (PUTH-REC-SOP-06-3.0-A27, # 2014003). The written consent was obtained from all participants involved. Clinical symptoms and radiological examination were used for the TOLF diagnosis as previously described [30]. We got TOLF and control ligaments from patients during the operation.

### Immunohistochemistry

Normal ligamentum flavum in adjacent levels from the same TOLF patient was used as the self-control group. TOLF tissues were fixed in 4% paraformaldehyde after carefully dehydrated and decalcified. The samples were embedded in paraffin, cut into 5-μm-thick sections and processed for immunohistochemistry. Immunohistochemistry was carried out by using standard protocols with the following antibodies: BMP2 (1:50, Abcam) and RUNX2 (1:50, Abcam). A computer-assisted genuine color image analysis system (Image-Pro Plus 6.0) was used to examine the in vivo expression of these markers in the TOLF tissues. Mean integrated optical densities were calculated as previously described [31].

### Cell culture

The primary cells of TOLF (OS) and ligamentum flavum control (Lig) were obtained from TOLF patients during surgery and washed with phosphate-buffered saline (PBS) as previously described [2]. The collected ligaments were minced and digested with 0.25% trypsin (Gibco, Grand Island, NY, USA) for 1 h, followed by 250U/mL type I collagenase (Sigma-Aldrich, St.Louis, MO, USA) for 4 h at 37˚C. The sample was put in 100mm dishes containing Dulbecco’s Modified Eagle’s medium (DMEM, Gibco) along with 10% fetal bovine serum (FBS, Gibco), 100U/mL penicillin G sodium and 100μg/mL streptomycin sulfate in a humidified atmosphere at 37°C. Passages 2 and 3 were used for the following related experiments.

### Flow cytometry

Flow cytometry was performed to determine the cell cycle phases of primary cultured cells as previously described [2]. Briefly, a total of 10^5^–10^6^ OS and Lig cells were obtained, washed three times with cold PBS, and fixed with 70% cold ethanol overnight. The cells were stained by 7-aminoactinomycin D (7-AAD) (20 μg/ml^-1^) (BD) with RNaseA (100 μg/ml^-1^) for no more than half an hour. The cells were then collected and filtered by 300 meshes. Flow cytometry analysis was carried out on a FACSAria II flow cytometer (BD Special Order System). Modfit LT 3.2 software (BD Special Order System) was selected to determine the percentage of phases of cell cycle of primary cultured cells.

### Alkaline phosphatase (ALP) staining

OS and Lig cells were seeded in 6-well plates at the density of 1×10^5^ /well and cultured in osteogenic medium as previously described [32]. Cells were fixed with 3.7% formaldehyde for 10 minutes at room temperature. ALP activity was then measured using an ALP activity staining kit (GENMED Scientifics, Shanghai, China) according to the manufacturer protocol.

### Alizarin red staining

OS and Lig cells were seeded in 6-well plates at the density of 1×10^5^/well and cultured in osteogenic medium as previously described [1]. Cells were washed with ice-cold PBS twice, and fixed with 90% cooled ethanol for 1 hr, then washed with water. Mineralization was evaluated using an Alizarin Red S kit (GENMED Scientifics, Shanghai, China) according to the manufacturer protocol. The red color obtained referred to calcium deposit.

### Enzyme-linked immunosorbent assay (ELISA)

Measurements of IL-6 and IL-1β were obtained from the disk supernatant of primary OS and Lig cell cultures. The amounts of IL-6 and IL-1β were determined by ELISA with a commercial Human IL-6 Quantikine ELISA Kit and Human IL-1β Quantikine ELISA Kit (R&D Systems, Minneapolis, MN, USA) according to the manufacturer protocol. 96-well ELISA microtiter plates (Corning, New York, NY, USA) were coated with goat anti-human IL-6 antibody or IL-1β antibody overnight at 4°C. The absorbance of each well at 325/420 nm was determined by a 2300 EnSpire^TM^ Multilabel Reader (PerkinElmer).

### Reverse transcription-quantitative PCR (RT-qPCR)

IL-6 (100 ng/ml; R&D Systems) was used to stimulate primary ligamentum flavum cells for 24 hr before harvest. TRIzol reagent (Invitrogen Life Technologies, Carlsbad, CA, USA) was used to extract total RNA from primary cultured cells as previously described [33]. The total RNA purity and integrity were determined using the RNA 6000 Nano assay with an Agilent Bioanalyzer 2100 (Agilent Technologies, Santa Clara, CA). 1μg total RNA was reverse transcribed into cDNA using the GoScript Reverse Transcription System (Promega Corp., Madison, WI, USA) according to the manufacturer protocol. qPCR was done in triplicate using SYBR-Green SuperReal PreMix Plus (Tiangen Biotech (Beijing) Co., Ltd., Beijing, China) and the iQ5 PCR system (Bio-Rad Laboratories, Inc., Hercules, CA, USA). The reaction conditions were used as follows: 95˚C for 30 sec, and 40 cycles of 95˚C for 10 sec and 60˚C for 30 sec. Data were represented as cycle threshold (Ct) values. The 2-^ΔΔ^Ct method was used to compare the RNA expressions. RNA levels were normalized to glyceraldehyde-3-phosphate dehydrogenase (GAPDH) levels. The primer sequences used are shown in Table1.

**Table 1:** List of the primer sequences for qRT-PCR assays.

Primer	Sequence (5’to3’)
Human GAPDH Forward	CAGGAGGCATTGCTGATGAT
Human GAPDH Reverse	GAAGGCTGGGGCTCATTT
Human ALP Forward	AAGGACGCTGGGAAATCTGT
Human ALP Reverse	GGGCATCTCGTTGTCTGAGT
Human BMP2 Forward	TCAAGCCAAACACAAACAGC
Human BMP2 Reverse	GGAGCCACAATCCAGTCATT
Human OSX Forward	GAGGTTCACTCGTTCGGATG
Human OSX Reverse	TGGTGTTTGCTCAGGTGGT
Human RUNX2 Forward	CCGTCCATCCACTCTACCAC
Human RUNX2 Reverse	ATGAAATGCTTGGGAACTGC
Human OCN Forward	CTCACACTCCTCGCCCTATT
Human OCN Reverse	CGCCTGGGTCTCTTCACTAC
Human CyclinD1 Forward	CCGTCCATGCGGAAGATC
Human CyclinD1 Reverse	GGAAGCGGTCCAGGTAGTTC
Human Cyclin C Forward	CCTTGCATGGAGGATAGTGAATG
Human Cyclin C Reverse	AAGGAGGATACAGTAGGCAAAGA

### Statistical analysis

All qPCR experiments were done in triplicate. Data were expressed as the mean ± SD and analyzed by SPSS version 12 statistical analysis package (SPSS Inc., Chicago, IL, USA). The measurements of TOLF thickness were compared using student’s t-test. *P* < 0.05 was considered statistically significant.

## Results

### Characterization of properties of TOLF

**Fig 1:**
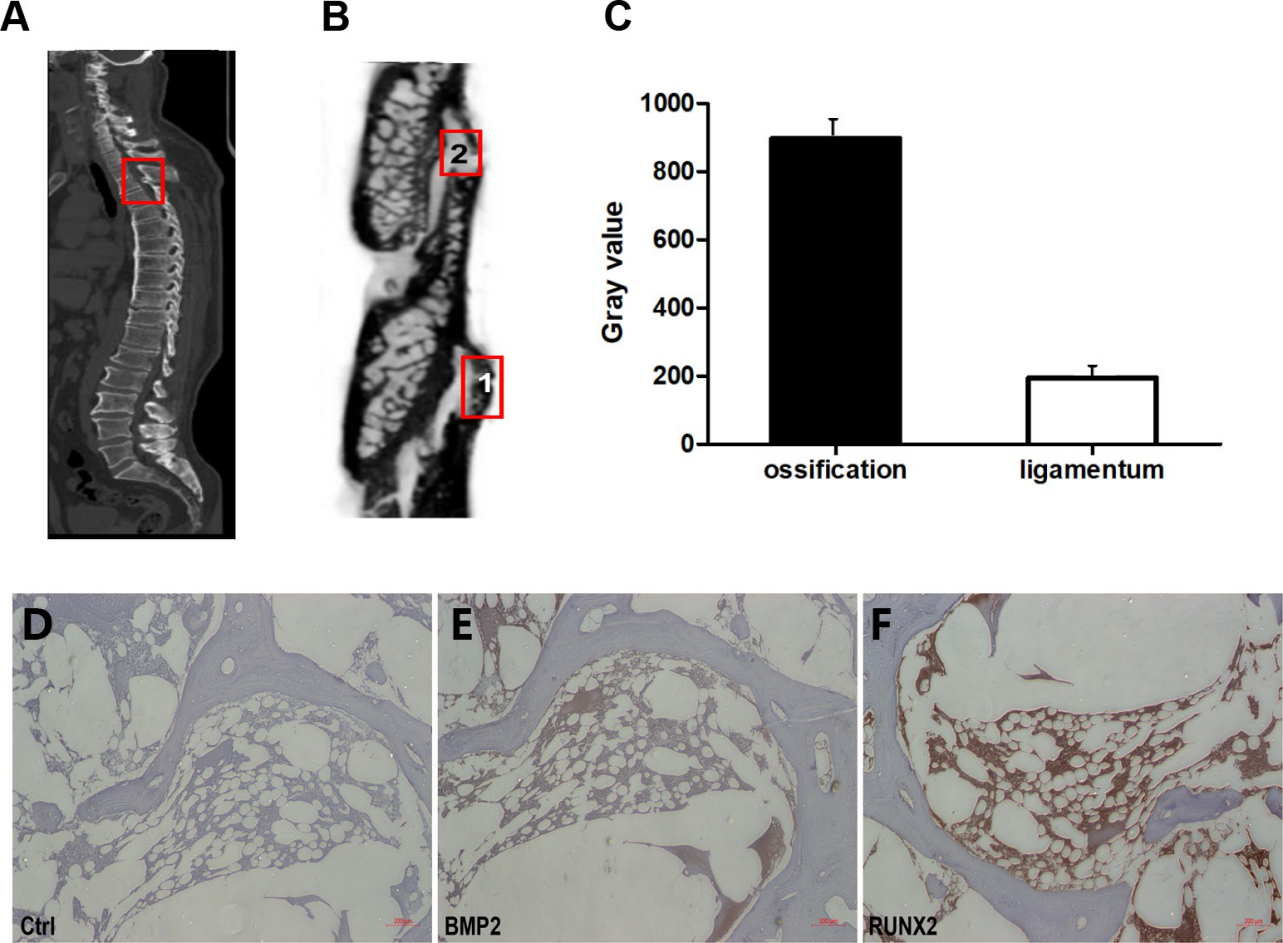
Characterization of properties of TOLF. A) CT image of TOLF patient. Ossified ligamentum flavum were observed. The thecal sac was compressed in TOLF; B) Micro-CT image of TOLF region after sagittal reconstruction. 1. ossified ligamentum flavum region with black color, 2. non-ossified ligamentum flavum region with white color; C) Gray value of red frame in B quantitation. Gray value represents different tissue density; (D–F), Immunohistochemical analysis of related markers expression levels in vivo in TOLF patients. Control (D, Ctrl); BMP2 (E) and RUNX2 (F).

Radiographic examinations were performed in TOLF patients involved. As shown in Fig 1A, the ossified intervertebral ligamentum flavum was observed in TOLF patient. The CT report showed that the spinal cord of TOLF patient was compressed. Micro-CT result of TOLF region was obtained after sagittal reconstruction. As showed in Fig 1B, the ossified ligamentum flavum region was labeled 1, and demonstrated high density, while the non-ossified ligamentum flavum region was labeled 2, and demonstrated in a low density. The tissue density was determined by quantitation of gray value in selected TOLF regions as showed in Fig 1C.

**Fig 2:**
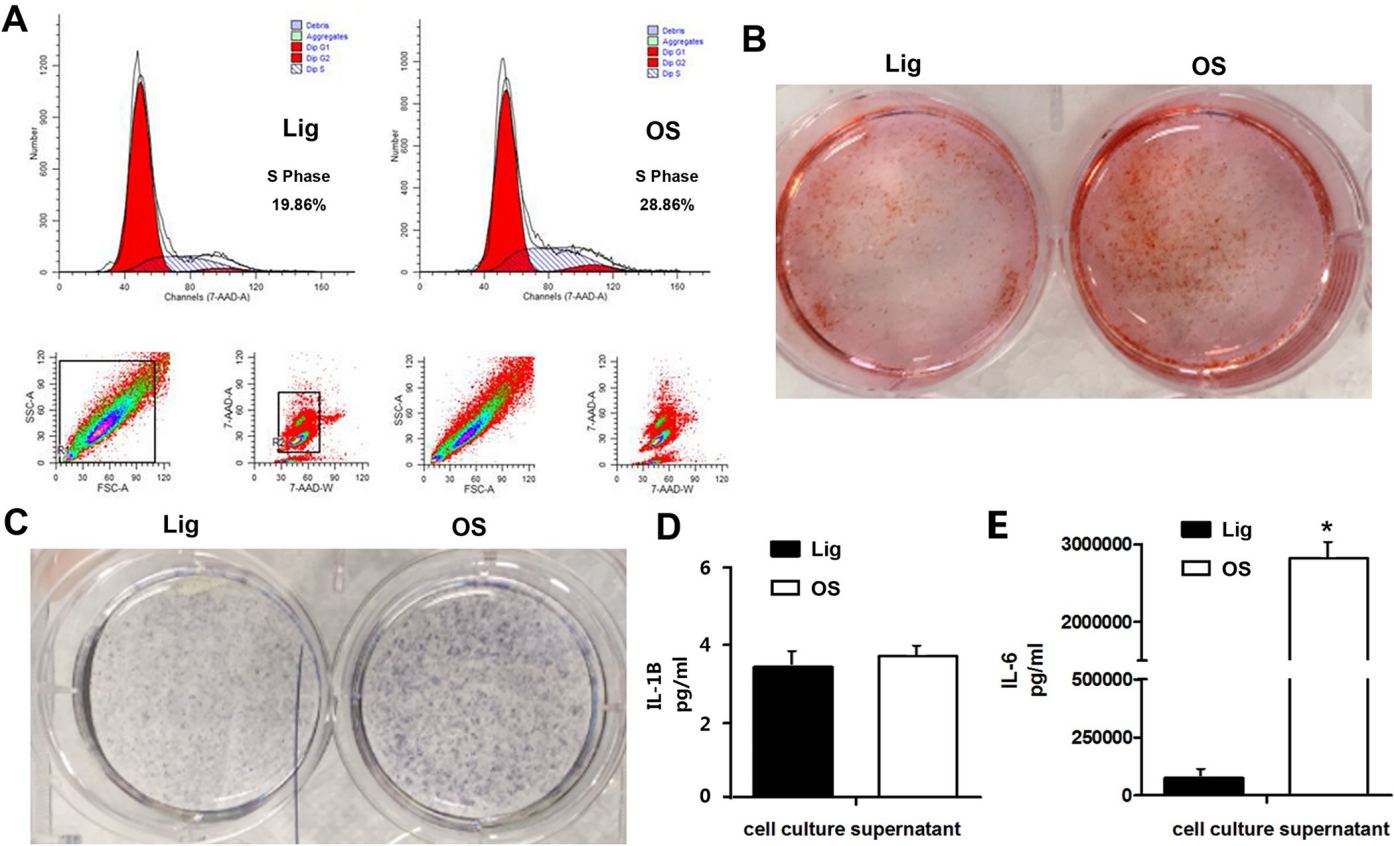
Comparison of OS cells and Lig cells from TOLF. (A) Cell cycle analysis by Flow cytometry. The proportion of S phase in OS cells was 9% higher than that in Lig cells; (B) Alizarin Red staining at day 18 showed calcification of OS was stronger than Lig; (C) Alkaline phosphatase (ALP) staining at day 18 showed that ALP activity of OS was higher than Lig; (D) ELISA for IL-1β expression levels in culture supernatant from OS and Lig cells; (E) ELISA for IL-6 expression levels in culture supernatant from OS and Lig cells.

Immunohistochemistry assays were carried out to examine the possible difference between the OS and Lig. Immunohistochemical analysis showed that osteoblastic BMP2 and RUNX2 protein were expressed in ossified areas of the ligament tissues from TOLF patient but could barely be detected in the normal ligaments from the same patient (Fig 1D–1F). Next, primary ligamentum flavum cells were isolated from the TOLF patients presenting with the OS or Lig. We have already observed that TOLF OS cells grew faster than Lig cells as previously described [2]. To further understand the difference in growth rate between OS and Lig cells, flow cytometry was performed to examine cell proliferation. As shown in Fig 2A, the fraction of S phase cells in OS cells was 28.86%, while that in Lig cells was 19.86%. Thus the proportion of S phase of cell cycle in primary cells isolated from ossified ligamentum flavum was 9% higher than the control. Furthermore, ALP staining and Alizarin red staining showed that TOLF OS were more ossified compared with the Lig control (Fig 2B and 2C), which were consistent with those of immunohistochemistry results.

### IL-6 promoted cell proliferation and ossification

Inflammatory cytokine is a potential factor associated with TOLF [2]. Our ELISA result showed that one of a inflammatory cytokines IL-6’s level in cell culture supernatant of OS was dramatically higher compared with the Lig control (Fig 2E), while another cytokine IL-1β‘s level remained unchanged (Fig 2D). These data led us to hypothesize that IL-6 is involved in the ligamentum ossification process in TOLF.

**Fig 3:**
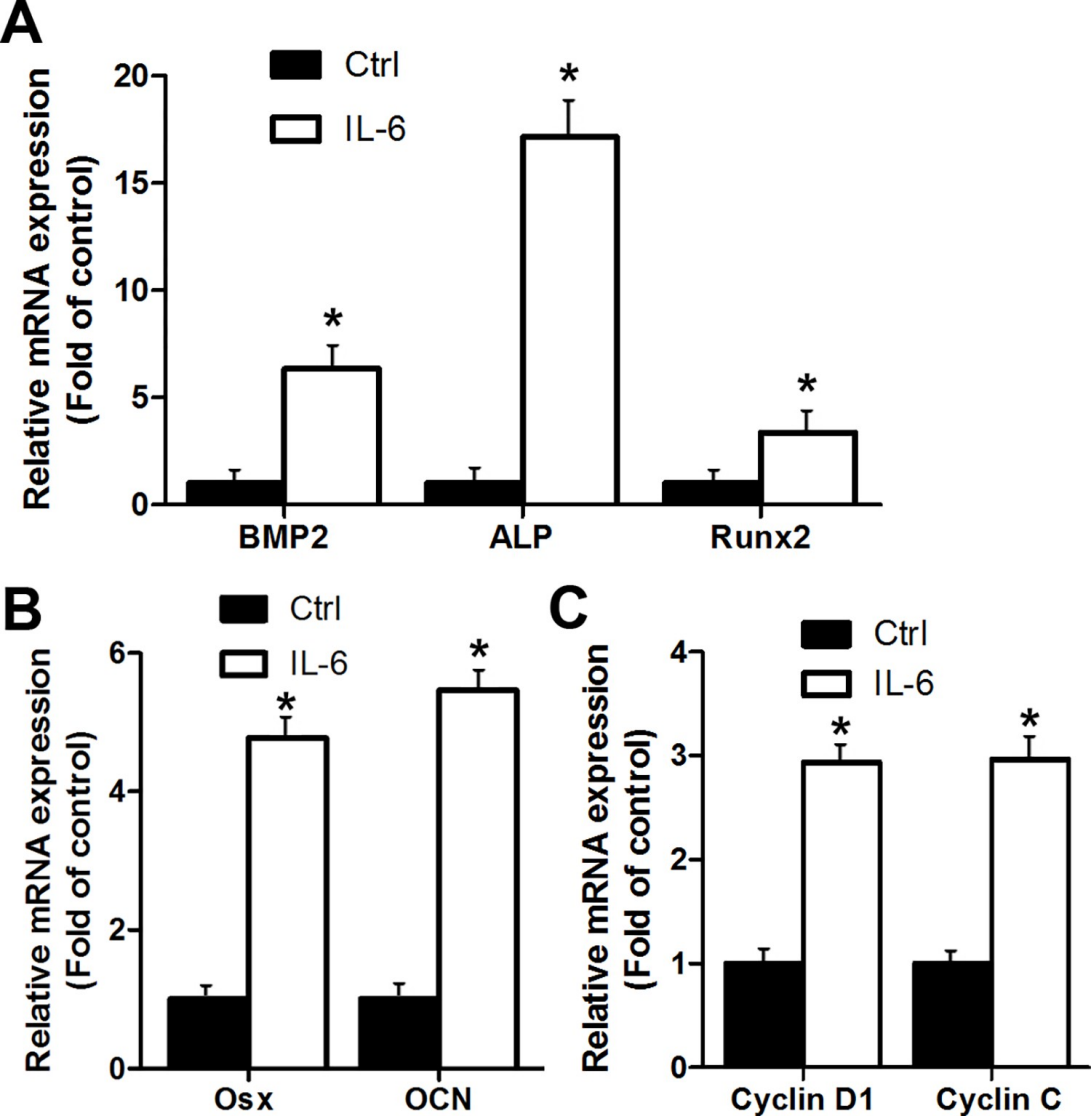
IL-6 stimulation enhances expressions of osteoblastic factors and cell proliferation factors in ligamentum flavum cells. (A) Effect of IL-6 on expressions of osteoblastic factors BMP2, ALP and RUNX2. Gene expressions were determined via qRT-PCR in ligamentum flavum cells with (IL-6) or without (Ctrl) IL-6 stimulation; (B) Effect of IL-6 on expressions of osteoblastic factors OSX and OCN. Gene expressions were determined via qRT-PCR in ligamentum flavum cells with (IL-6) or without (Ctrl) IL-6 stimulation; (C) Effect of IL-6 on expressions of cell proliferation factors cyclin D1 and cyclin C. Gene expressions were determined via qRT-PCR in ligamentum flavum cells with (IL-6) or without (Ctrl) IL-6 stimulation.

To test our hypothesis that IL-6 is involved in the ligamentum ossification process in TOLF, the effects of IL-6 on expressions of both osteoblastic factors and cell proliferation factors were examined respectively. Primary ligamentum flavum cells were stimulated by 100 ng/ml of IL-6 for 24 hr followed by cell lysis. Total RNA was extracted from cultured cells and analyzed by RT-qPCR. As shown in Fig 3, IL-6 stimulation activated expressions of osteoblastic factors BMP2, ALP and RUNX2 (Fig 3A) as well as OSX and OCN (Fig 3B). ALP and OCN are well-known downstream osteoblastic genes of RUNX2 and OSX. Expressions of cell cycle related genes such as cell proliferation factors cyclin D1 and cyclin C were also increased (Fig 3C) after IL-6 stimulation. These observations suggest that IL-6 promoted cell proliferation and ossification

### IL-6 regulated the osteogenic BMP2 activation through p38 MAPK pathway

**Fig 4:**
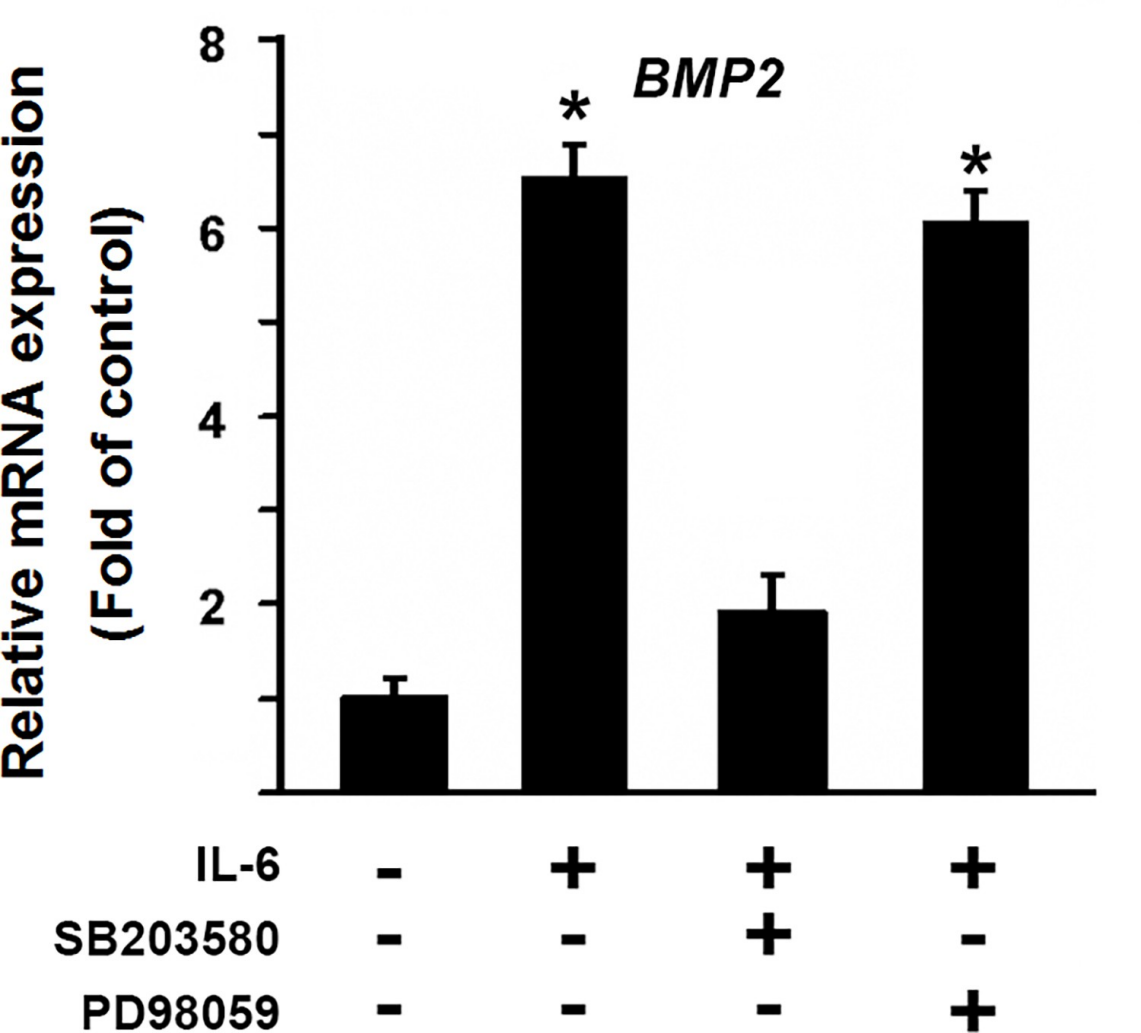
IL-6-induced BMP2 activation is mediated through p38 MAPK pathway. Gene expressions were determined via qRT-PCR in primary ligamentum flavum cells with or without IL-6 stimulation. Specific inhibitors (10μM SB203580 or 50μM PD98059) were added as indicated.

We were interested in through which pathway IL-6 regulated osteoblastic factor BMP2. We applied primary ligamentum flavum cells to address this question. Loss-of-function approach was used to examine possible pathways involved. SB203580 as a specific inhibitor for p38 MAPK pathway was selected for the assay. PD98059 is a specific ERK inhibitor in mitogen-activated protein kinase pathway. Inhibitors were added in the culture medium as indicated. As showed in Fig 4, IL-6 stimulation activated BMP2 expression by 6.5 fold. Addition of 10 μM SB203580 almost abolished the activation of BMP2 expression induced by IL-6. However, IL-6-induced BMP2 activation did not change after the treatment with 50 μM PD98059. These findings suggested that IL-6 regulated the osteogenic BMP2 activation through p38 MAPK pathway.

## Discussion

TOLF is a disease mostly within East Asia, and results in high morbidity in patients over the age of 65. Surgery is the only clinical approach available so far to treat TOLF. The underlying mechanisms of TOLF progress are not clear, which hampers to develop new potential treatment strategies. Some research groups have studied possible association of cytokines and transcriptional factors such as BMP2/4, prostaglandin I2, endothelin and RUNX2 with TOLF [34,35]. Inflammatory factor has been considered to be one of possible causes of TOLF. Our recent studies have identified inflammation factors such as TNF-α involved in ossified ligamentum flavum in TOLF, and we found that inflammatory factor stimulation led to the elevated expressions of osteoblastic genes, such as BMP2, OCN,ALP, etc [1,2]. The purpose of this study is to further characterize the properties of TOLF and to continue to explore its underlying mechanism.

We have already observed that TOLF OS cells grew faster than Lig cells [2]. In this study, flow cytometry was carried out to determine the cell cycle phases of primary TOLF cells, and our results in Fig 2A demonstrated that the proportion of S phase of cell cycle in OS cells was 9% higher than the control. This may partially explain faster growth of OS cells as we observed previously [2]. Alizarin red staining and ALP staining were consistent with immunohistochemistry results of increased expressions of osteoblastic BMP2 and RUNX2 proteins. These results confirm the clinical observation of ossification of the ligamentum flavum in TOLF. We also found that IL-6 level dramatically increased in the culture supernatant of OS cells while IL-1β‘s level remained unchanged. It has been reported that IL-6 participates in conducting cell proliferation, differentiation between mesenchymal stem cells and neuronal cells [36]. We addressed the effect of IL-6 on the ligamentum ossification process in TOLF in this study.

Our data indicated that IL-6 promoted cell proliferation and ossification. Both cell proliferation and differentiation are important during bone formation. In this study, we demonstrated that the proportion of S phase of cell cycle in OS cells was higher (Fig 2A). In addition, our results showed that cell proliferation factors cyclin D1 and cyclin C were upregulated after IL-6 stimulation (Fig 3C). These data support our previous observation that TOLF OS cells grew faster than Lig cells [2]. On the other hand, our observations provide evidence that IL-6 activated the expressions of osteoblastic genes, including BMP2, ALP, RUNX2, OSX and OCN (Fig 3). This is consistent with another study showing that addition of IL-6 favors osteoblastic differentiation [37]. It has been reported that inflammatory factor stimulation induced osteoblastogenesis through activating cell signaling pathways such as ERK1/2 and p38 MAPK [38]. Once activated, these signals promote the expression and activation of key osteogenic factors, such as BMP2 and RUNX2 [39]. This led us to address the possibility that IL-6 regulated the osteogenic activity via ERK1/2 or p38 MAPK signaling pathway. Indeed, loss-of-function approach using p38 MAPK inhibitor SB203580 in Fig 4 showed that SB203580 was able to inhibit IL-6-induced activation of the osteogenic BMP2 expression. These findings suggest that p38 MAPK pathway was responsible in part for mediating the osteogenic BMP2 activation by IL-6. However, the inhibition of p38 MAPK pathway did not achieve the full blockade of IL-6-induced osteogenic BMP2 activation, so this cannot rule out other possible mechanisms for IL-6’s effect on osteogenic BMP2 activation. Therefore, further research is needed to better understand the mechanisms of the effect of IL-6 on ligamentum flavum ossification in TOLF. Although IL-6’s level in cell culture supernatant of OS was dramatically higher (Fig 2E), while IL-1’s level remained unchanged (Fig 2D), the data cannot rule out the possibility that other cytokines may also participate in TOLF. This deserves further investigation.

In conclusion, this study further characterized the properties of TOLF to confirm the clinical observation of ossification of the ligamentum flavum in TOLF. Our new findings suggest IL-6 is involved in the ligamentum ossification process in TOLF.

## Abbreviations

TOLF: Thoracic ossification of the ligamentum flavum
IL-6: interleukin-6
BMP2: bone morphogenetic protein-2
OSX: Osterix
OCN: osteocalcin
ALP: Alkaline phosphatase
GAPDH: glyceraldehyde 3 phosphate dehydrogenase
